# Epidemiological, Clinical, and Trichoscopic Features of Syphilitic Alopecia: A Retrospective Analysis and Systematic Review

**DOI:** 10.3389/fmed.2022.890206

**Published:** 2022-05-02

**Authors:** Cherrin Pomsoong, Siriorn Sukanjanapong, Yanisa Ratanapokasatit, Poonkiat Suchonwanit

**Affiliations:** Division of Dermatology, Department of Medicine, Faculty of Medicine, Ramathibodi Hospital, Mahidol University, Bangkok, Thailand

**Keywords:** alopecia, alopecia syphilitica, dermoscopy, hair loss, moth-eaten, syphilis, treponema, trichoscopy

## Abstract

**Background:**

Syphilitic alopecia (SA), which mimics other types of alopecia, is an uncommon manifestation of secondary syphilis. Trichoscopic features may facilitate its diagnosis. However, studies on SA and its trichoscopic characteristics remain limited.

**Objective:**

To investigate the epidemiological, clinical, and trichoscopic findings and laboratory results, treatment, and outcomes of SA in Thai patients as well as to comprehensively summarize all trichoscopic features of SA through a systematic review.

**Methods:**

Data on patients diagnosed with SA between December 2010 and December 2021 were obtained from their medical records and analyzed retrospectively. A systematic review of trichoscopic data, both from our institution and from studies registered in the PubMed, MEDLINE, and Embase databases, was conducted. A descriptive summarization was performed to comprehensively study the trichoscopic features of SA.

**Results:**

Of the 205 patients with secondary syphilis, 23 patients with SA (symptomatic SA: 20, essential SA: 3) were included. The mean age was 27.6 ± 8.8 years, and male predominance was noted. The moth-eaten pattern was the most common SA presentation, and the parieto-occipital scalp was the most commonly affected area. All patients with SA achieved significant hair regrowth within 3 months of antibiotic therapy. Trichoscopic images were available for 20 patients with SA from our institute and were included in the systematic review. Fourteen articles provided information on 21 patients. Overall (*N* = 41), 26 (63.4%), 8 (19.5%), and 7 (17.1%) patients had moth-eaten alopecia, diffuse alopecia, and mixed alopecia, respectively. The most frequent trichoscopic finding was short regrowing hairs (78%), followed by decreased hair per follicular unit (75.6%), and empty follicles (51.2%). Unique features included flame hairs, bent tapering hairs, reddish-brown background, and brown rings around the perifollicular areas, each described in one case. However, the results were based only on case reports and small case series.

**Conclusions:**

Given the progressively increasing frequency of SA, trichoscopic examination may be valuable when SA is suspected in patients with idiopathic alopecia; however, our findings are quite non-specific. The absence of exclamation mark hairs may help in the diagnosis of SA. Further comparative studies on other types of alopecia are required to determine the most useful diagnostic features.

## Introduction

Syphilitic alopecia (SA) is an uncommon non-scarring hair loss in secondary syphilis, with a prevalence ranging from 2.9 to 22.2% (1–3). SA is generally recognized as a great imitator owing to its various manifestations that mimic other hair disorders, such as alopecia areata, trichotillomania, tinea capitis, and telogen effluvium (3). It can manifest three clinical patterns that affect not only the scalp region, but also other hair-covered areas; these patterns include moth-eaten, diffuse, and mixed alopecia (4). In 1940, McCarthy described two types of SA based on the associated cutaneous presentations, and this classification continues to be used to date. These types comprise symptomatic SA (which presents with mucocutaneous lesions of secondary syphilis) and essential SA (which is characterized by alopecia alone, i.e., without other syphilitic lesions) (2).

The cutaneous features of secondary syphilis may help in the diagnosis of SA; however, they are absent in patients with essential SA. Given the progressive rise in the number of cases of SA in recent years, misdiagnosis and underestimation still occur frequently (5). Trichoscopy is increasingly used as a non-invasive tool for the diagnosis of hair and scalp disorders (6–8). Despite the presence of cutaneous signs and laboratory findings, trichoscopic abnormalities may represent the specific features of SA and help differentiate it from other causes of alopecia (9–11).

Currently, due to the rarity of SA, there is limited information on its epidemiological, clinical, and trichoscopic characteristics. This study aimed to investigate the prevalence, clinical and trichoscopic findings, laboratory results, treatment, and outcomes of SA in Thai patients and comprehensively summarize all trichoscopic features of SA using a systematic review of cases from our institute and the medical literature.

## Methods

### Study Design

This retrospective study was conducted at the Ramathibodi Hospital, Bangkok, Thailand, in accordance with the principles of the Declaration of Helsinki. The Mahidol University Institutional Review Board for Ethics in Human Research (MURA2022/99) approved this study. The requirement for informed consent was waived, and the data were anonymized prior to analysis.

### Patients

We retrospectively reviewed the medical records of patients diagnosed with secondary syphilis between December 2010 and December 2021. The diagnosis was established in accordance with the Center of Disease Control criteria (12, 13). Of all secondary syphilis cases, the diagnosis of SA was based on the following: (i) hair loss within 6 months after disease onset and significant (cosmetically acceptable) hair regrowth within 6 months after antibiotic therapy; and/or (ii) histopathological features compatible with SA. Furthermore, eligible patients were required to have positive serological tests for reagin and anti-treponemal antibodies. Patients with clinical and investigational findings inconsistent with secondary syphilis or SA, other concomitant hair and scalp disorders, and incomplete medical records were excluded.

### Data Collection and Assessment

Data on patient characteristics, including age, sex, disease duration, comorbidities, clinical manifestations, laboratory investigation, treatment regimen, and outcomes were extracted. The clinical features of SA included moth-eaten alopecia (localized non-scarring alopecic patch/patches with irregular borders), diffuse alopecia (uniform hair loss over the entire scalp), and mixed pattern (combination of moth-eaten and diffuse alopecia). Alopecia of the eyebrows or other body areas was also recorded. Trichoscopic images were reviewed and re-evaluated by a hair specialist (PS). Data regarding the epidemiology, clinical and trichoscopic features, laboratory results, treatment, and outcomes of SA in Thai patients are presented separately.

### Systematic Review

We further conducted a systematic review on the clinical and trichoscopic features of SA following the guidelines of the “Preferred Reporting Items for Systematic Reviews and Meta-Analyses” (PRISMA) checklist for qualitative assessment. Two authors (CP and SS) independently performed literature searches in the PubMed, MEDLINE and Embase databases for articles published from each database's inception to February 2022. The search keywords included “syphilitic alopecia,” “alopecia syphilitica,” “alopecia,” “dermoscopy,” “dermatoscopy,” “trichoscopy,” and “syphilis.” We limited the preliminary searches to articles in the English language. The reference lists of the retrieved articles were then assessed and additionally hand-searched.

The search results from all databases were aggregated and duplicates were removed. All articles reporting trichoscopic features of SA were included and independently reviewed by two authors (CP and SS). The quality and risk of bias of the included studies were assessed using a tool to evaluate the methodological quality of the case reports and case series. Data were extracted on the publication year, patient demographics, and clinical and trichoscopic characteristics. The available trichoscopic images in the included articles were reassessed. If the two authors failed to reach an agreement, an independent third author (PS) was summoned for discussion and making the final decision. Data on the clinical characteristics and reported signs visualized on trichoscopy, both from our institute and the previous literature, were combined and analyzed. This systematic review was registered in the PROSPERO database (ID 313791).

### Statistical Analysis

The demographics, clinical and trichoscopic characteristics, laboratory results, treatment, and outcomes have been presented in the descriptive analyses. Categorical data are presented as numbers and proportions. Continuous variables are expressed as means (standard deviations) and as medians (interquartile ranges) for normally and non-normally distributed data, respectively. In the pooled analysis, we combined data from our institute and the literature, and missing data were recorded as “not reported.” Chi-square and Fisher's exact tests were used to compare trichoscopic features between groups as appropriate. A *P*-value < 0.05 was considered statistically significant. All analyses were performed using STATA Data Analysis, version 14.1 (StataCorp, College Station, TX, USA).

## Results

### Syphilitic Alopecia in the Thai Population

Two hundred and five patients were diagnosed with secondary syphilis; 11 were excluded due to incomplete medical records. Of the remaining 194 patients, 156 reported no associated alopecia and 15 presented with hair loss that did not match the diagnostic criteria of SA. The demographic and clinical characteristics of the patients with SA at our institute are summarized in Table 1. SA was observed in 23 (11.2%) patients; these comprised 20 and three patients with symptomatic and essential SA, respectively. The mean age was 27.6 ± 8.8 years, and a male predominance was observed (*n* = 20, 87%). The median disease duration was 1 (1–3) month. We noted that 34.8% (*n* = 8), 21.7% (*n* = 5), and 21.7% (*n* = 5) of the patients had unprotected sex, multiple partners, and comprised men who had sex with men, respectively. The serological tests were positive for reagin and antitreponemal antibodies in all patients. Approximately 50% of the patients (*n* = 13, 56.5%) were positive for the human immunodeficiency virus, with a median CD4 count of 199 (37–343) cells/μL and a median viral load of 51 (40–11,000) copies/mL.

**Table 1:** Dermographics, clinical characteristics, laboratory results, and treatment of Thai patients with syphilitic alopecia.

**Demographics**	**(***N*** = 23)**
**Gender,** ***n*** **(%)**
∙ Male	20 (87)
∙ Female	3 (13)
**Age of onset, years, mean ±SD**	27.6 ± 8.8
**Duration of onset, months, median (IQR)**	1 (1, 3)
**STD-related risks,** ***n*** **(%)**
∙ Unprotected sex	8 (34.8)
∙ Multiple partners	5 (21.7)
∙ Men who have sex with men	5 (21.7)
**Baseline VDRL titer,** ***n*** **(%)**
∙>1:64	3 (13)
∙ 1:32–1:64	19 (82.6)
∙ 1:8–1:16	1 (4.4)
**Concomitant infection,** ***n*** **(%)**
∙ HIV infection	13 (56.5)
∙ HBV infection	2 (8.7)
**Laboratory results in HIV, median (IQR)**
∙ CD4 count, cells/μL	199 (37–343)
∙ Viral load, copies/mL	51 (40–11,000)
**Clinical characteristics**
**Type of syphilitic alopecia,** ***n*** **(%)**
∙ Symptomatic	20 (87)
∙ Essential	3 (13)
**Pattern of syphilitic alopecia,** ***n*** **(%)**
∙ Moth-eaten pattern	13 (56.5)
∙ Diffuse pattern	4 (17.4)
∙ Mixed pattern	6 (26.1)
**Affected scalp area,** ***n*** **(%)**
∙ Parieto-occipital area	19 (82.6)
∙ Vertex area	2 (8.7)
**Alopecia of the eyebrows**	4 (17.4)
**Mucocutaneous manifestations,** ***n*** **(%)**
∙ Maculopapular rash	14 (60.9)
∙ Papulosquamous lesions of the palms and/or soles	12 (52.2)
∙ Condyloma lata	4 (17.4)
**Neurosyphilis,** ***n*** **(%)**	4 (17.4)
**Treatment,** ***n*** **(%)**
∙ Benzathine penicillin G	19 (82.6)
∙ Doxycycline	4 (17.4)

*HBV, hepatitis B virus; HIV, human immunodeficiency virus; IQR, interquartile range; SD, standard deviation; STD, sexually transmitted disease; VDRL, venereal disease research laboratory*.

**Figure 1:**
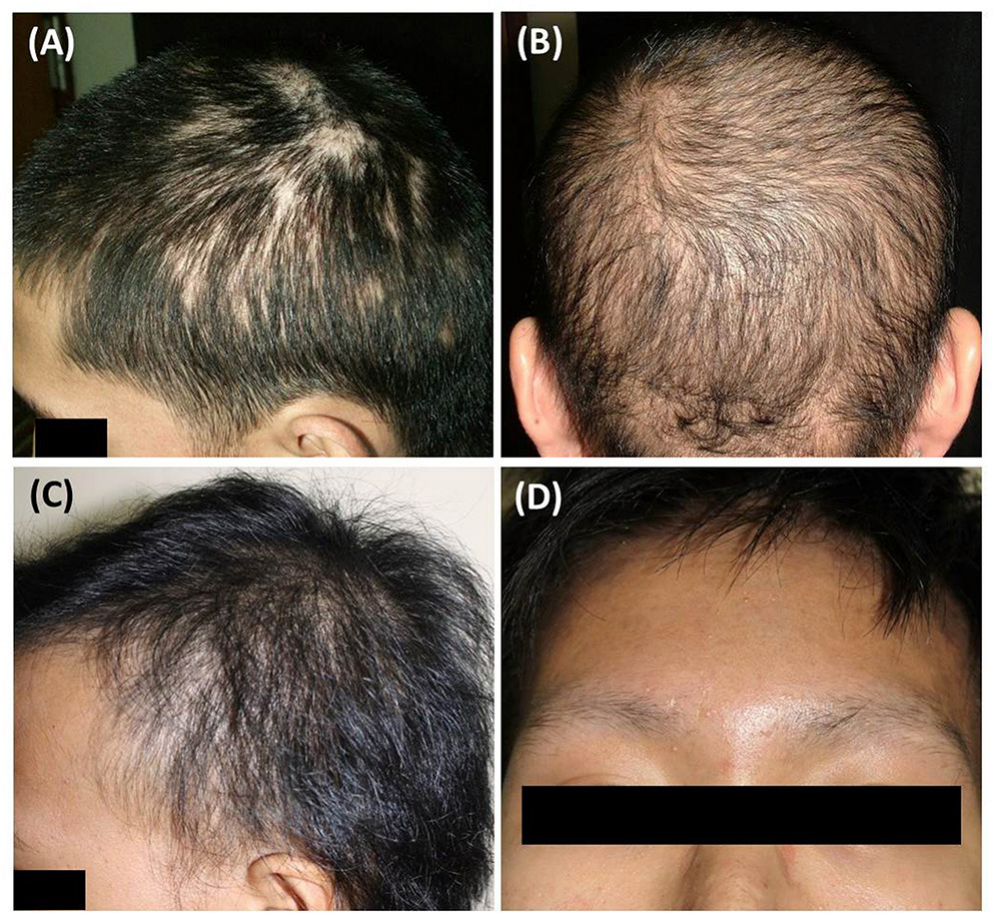
Clinical manifestations of syphilitic alopecia: **(A)** moth-eaten alopecia; **(B)** diffuse alopecia; **(C)** mixed pattern; **(D)** alopecia of the eyebrows.

Clinically, the moth-eaten pattern was the most common SA presentation (*n* = 13, 56.5%; Figure 1A), followed by the mixed pattern (*n* = 6, 26.1%; Figure 1C) and diffuse alopecia (*n* = 4, 17.4%; Figure 1B). Among the scalp regions, the parieto-occipital region and vertex were affected in 82.6% (*n* = 19) and 8.7% (*n* = 2) of the patients, respectively. Associated eyebrow loss was reported in four patients (17.4%; Figure 1D). Scalp biopsy for histopathological diagnosis was performed in 16 (69.5%) patients, because the clinical and trichoscopic features could not be distinguished from those of other hair disorders.

**Figure 2:**
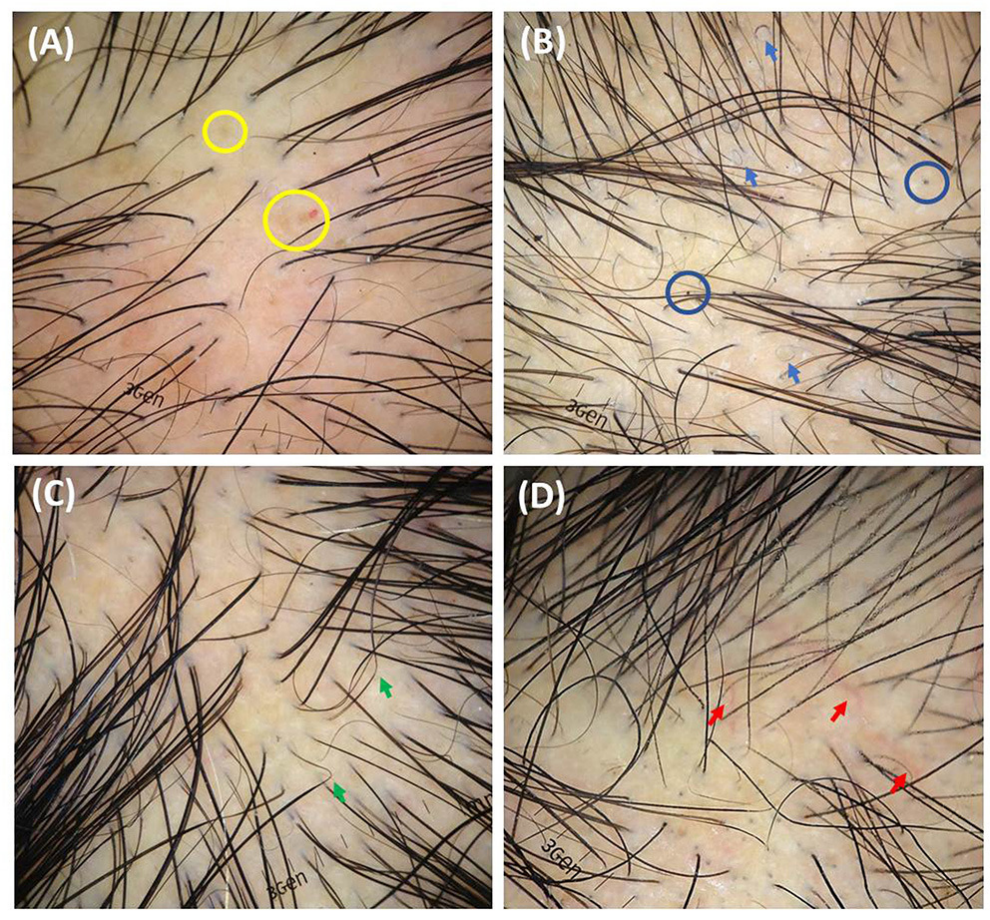
Trichoscopic features of syphilitic alopecia (original magnification x20): **(A)** decreased hair per follicular unit, empty follicles, yellow dots (yellow circles), and erythematous background; **(B)** short regrowing hairs, pigtail hairs (blue arrows), broken hairs, black dots (blue circles), perifollicular and interfollicular scales; **(C)** zigzag hairs (green arrows), black dots, and empty follicles; **(D)** decreased hair per follicular unit, black dots, and telangiectasia (red arrows).

Regarding the trichoscopic features (Table 2), trichoscopic images were available for 20 patients in our database (Figures 2A–D). The most common presentations of the moth-eaten pattern were empty follicles (8/10), followed by short regrowing hairs (7/10), decreased hair per follicular unit (6/10), and an erythematous background (6/10). The most common presentations of the diffuse pattern included decreased hair per follicular unit (4/4) and short regrowing hairs (4/4) while yellow dots (5/6) were the most frequent trichoscopic finding of the mixed pattern. There was no statistically significant difference in trichoscopic features among the three patterns of SA (all *P* ≥ 0.05).

**Table 2:** Trichoscopic findings of Thai patients with syphilitic alopecia.

**Trichoscopic features**	**Pattern of syphilitic**			***P*** **-value**
	**alopecia,** ***n*** **(%)**			
	**Moth-eaten** **pattern** **(***n*** = 10)**	**Diffuse** **pattern** **(***n*** = 4)**	**Mixed** **pattern** **(***n*** = 6)**	
**Hair shaft changes**				
∙ Short regrowing hairs	7 (70)	4 (100)	3 (50)	0.548
∙ Decreased hair per follicular unit	6 (60)	4 (100)	3 (50)	0.585
∙ Hypopigmented hair shafts	3 (30)	2 (50)	1 (16.7)	0.813
∙ Broken hairs	1 (10)	1 (25)	2 (33.3)	0.508
∙ Zigzag hairs	1 (10)	0	0	-
∙ Pigtail hairs	0	1 (25)	1 (16.7)	0.724
**Scalp surface changes**				
∙ Empty follicles	8 (80)	2 (50)	3 (50)	0.371
∙ Yellow dots	4 (40)	1 (25)	5 (83.3)	0.131
∙ Black dots	3 (30)	1 (25)	2 (33.3)	0.961
∙ Erythematous background	6 (60)	1 (25)	2 (33.3)	0.389
∙ Telangiectasia	1 (10)	0	0	–
∙ Perifollicular scales	1 (10)	1 (25)	0	0.769
∙ Interfollicular scales	1 (10)	1 (25)	0	0.769

Maculopapular eruption and papulosquamous lesions of the palms and soles were observed in 14 (60.9%) and 12 (52.2%) patients, respectively. Neurosyphilis was observed in four cases (17.4%). Most patients with SA had baseline VDRL titers ≥1:32 (*n* = 22, 95.6%) and were treated with intramuscular benzathine penicillin G (*n* = 19, 82.6%) and doxycycline (*n* = 4, 17.4%). All patients with SA achieved significant hair regrowth within 6 months after the end of therapy, and the serological response was adequate.

### Systematic Review of Clinical and Trichoscopic Features of Syphilitic Alopecia

**Figure 3:**
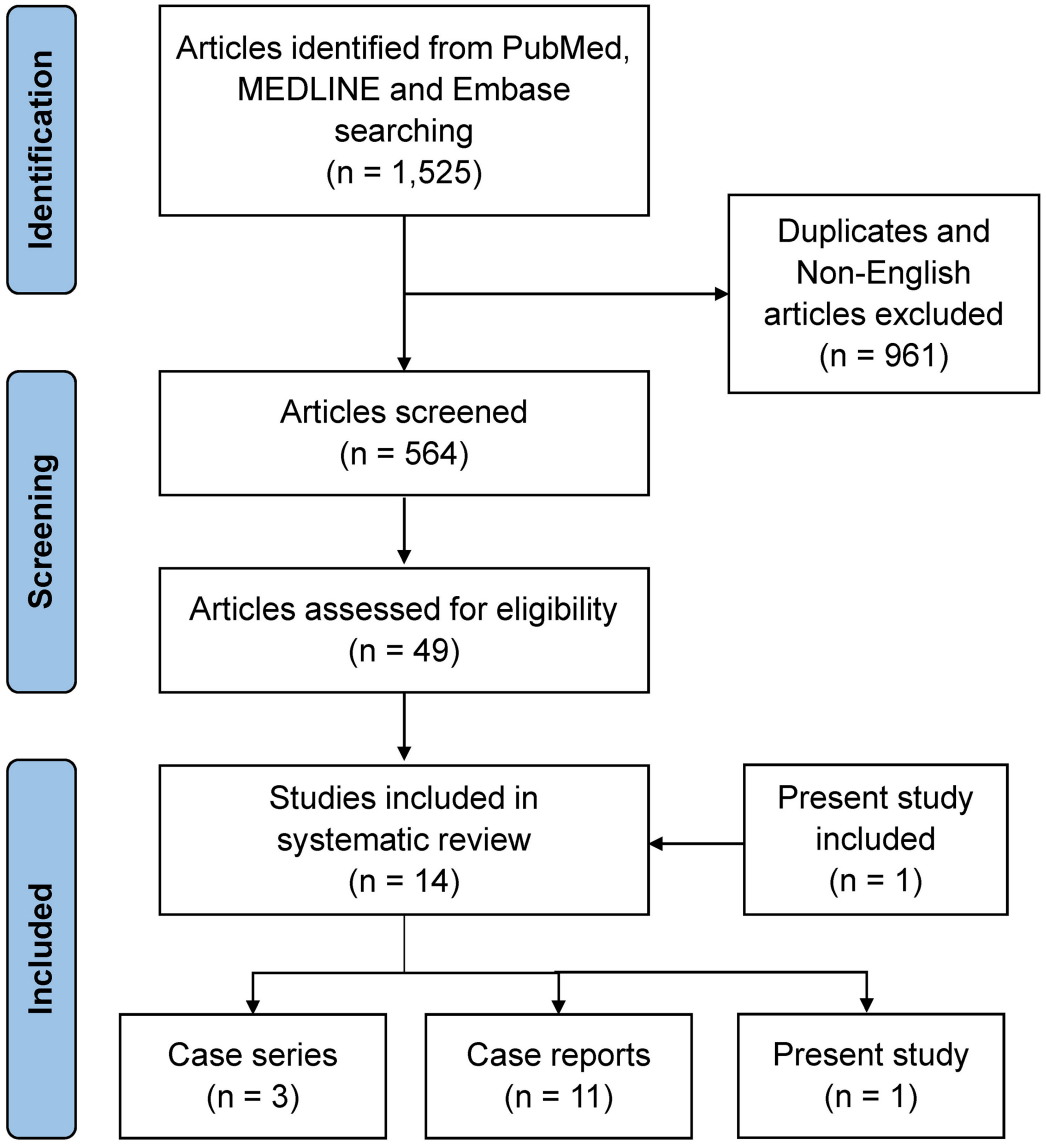
Flow diagram of study selection based on the Preferred Reporting Items for Systematic Reviews and Meta-analyses (PRISMA) flowchart for the article selection process.

We identified 1,525 articles on SA in the online databases. A total of 564 studies remained after duplicates and non-English articles were excluded. After screening and assessment for eligibility, 14 studies (including three case series and 11 case reports) were included in the systematic review (2, 9, 10, 14–24). The 14 included studies reported 21 patients diagnosed between 2014 and 2021. The results of the methodological quality and risk of bias assessment revealed that the most significant drawback in the included studies was the absence of a clear selection method. A flow diagram, created according to the PRISMA guidelines, is illustrated in Figure 3, and the characteristics of the included studies are shown in Table 3.

**Table 3:** Characteristics of studies included in the systematic review.

**Year**	**References**	**Country**	**Number of cases (*n*)**	**Sex (** ***n*** **)**		**Mean age (years)**	**Type of syphilitic alopecia (** ***n*** **)**		**Pattern of syphilitic alopecia (** ***n*** **)**		
				**Male**	**Female**		**Symptomatic**	**Essential**	**Moth-eaten**	**Diffuse**	**Mixed**
2014	Ye et al. (9)	China	1	1	0	42	0	1	1	0	0
2015	Piraccini et al. (14)	Italy	5	5	0	38	4	1	4	1	0
2016	Fukui et al. (15)	Japan	1	1	0	46	1	0	1	0	0
2017	Doche et al. (16)	Brazil	3	3	0	41	2	1	2	1	0
2017	Tognetti et al. (2)	Italy	1	1	0	34	1	0	0	0	1
2018	Costa et al. (17)	Italy	2	1	1	19	0	2	0	2	0
2018	Sebaratnum and Wong (18)	Australia	1	1	0	35	1	0	1	0	0
2019	Li and Wu (19)	China	1	1	0	19	1	0	1	0	0
2020	Bomfim et al. (10)	Brazil	1	1	0	29	1	0	1	0	0
2021	Ciupińska et al. (20)	Poland	1	1	0	22	1	0	1	0	0
2021	Gomes et al. (21)	Portugal	1	1	0	20	1	0	1	0	0
2021	Vaccaro et al. (22)	Italy	1	1	0	39	0	1	1	0	0
2021	Lin et al. (23)	China	1	1	0	22	1	0	1	0	0
2022	Kosidcanasup and Vejjabhinanta (24)	Thailand	1	1	0	31	0	1	1	0	0
2022	Present study	Thailand	20	18	2	30.2	17	3	10	4	6

After pooling information from our cohort (*n* = 20) and all enrolled studies (*n* = 21), 41 patients with SA with trichoscopic findings were evaluated, and the results are shown in Table 4. The mean age was 31.6 ± 8.9 years, and a male predominance was observed (*n* = 38, 92.7%). The median time to SA presentation was 2 (1, 2) months. Symptomatic and essential SA were reported in 75.6% (*n* = 31) and 24.4% (*n* = 10) of the patients, respectively. Moth-eaten alopecia was the most common pattern (*n* = 26, 63.4%), followed by diffuse (*n* = 8, 19.5%), and mixed alopecia (*n* = 7, 17.1%).

**Table 4:** Pooled analysis of present study and the literature regarding demographics, clinical characteristics, and trichoscopic features of syphilitic alopecia.

**Demographics and clinical characteristics (*N* = 41)**
**Gender,** ***n*** **(%)**
∙ Male	38 (92.7)
∙ Female	3 (7.3)
**Age of onset, years, mean** **±SD**	31.6 ± 8.9
**Duration of alopecia, months, median (IQR)**	2 (1, 2)
**Type of syphilitic alopecia,** ***n*** **(%)**
∙ Symptomatic	31 (75.6)
∙ Essential	10 (24.4)
**Pattern of syphilitic alopecia,** ***n*** **(%)**
∙ Moth-eaten pattern	26 (63.4)
∙ Diffuse pattern	8 (19.5)
∙ Mixed pattern	7 (17.1)
**Trichoscopic features,** ***n*** **(%)**
∙ Short regrowing hairs	32 (78)
∙ Decreased hair per follicular unit	31 (75.6)
∙ Empty follicles	21 (51.2)
∙ Yellow dots	18 (43.9)
∙ Erythematous background	18 (43.9)
∙ Black dots	14 (34.1)
∙ Broken hairs	12 (29.3)
∙ Perifollicular scales	5 (12.2)
∙ Interfollicular scales	4 (9.6)
∙ Telangiectasia	4 (9.6)
∙ Hypopigmented hair shafts	2 (4.9)
∙ Pigtail hairs	2 (4.9)
∙ Zigzag hairs	2 (4.9)
∙ Flame hairs	1 (2.4)
∙ Bent tapering hairs	1 (2.4)
∙ Brown rings around perifollicular areas	1 (2.4)
∙ Reddish-brown background	1 (2.4)

*IQR, interquartile range; SD, standard deviation*.

Extracted data on the trichoscopic findings of SA from our cohort and the included articles are summarized in Table 4. Short regrowing hairs was the most common feature (*n* = 32, 78%), followed by decreased hair per follicular unit (*n* = 31, 75.6%), empty follicles (*n* = 21, 51.2%), yellow dots (*n* = 18, 43.9), an erythematous background (*n* = 18, 43.9%), black dots (*n* = 14, 34.1%), and broken hairs (*n* = 12, 29.3%). Less-frequently reported findings included hypopigmented hair shafts (*n* = 2, 4.9%), pigtail hairs (*n* = 2, 4.9%), zigzag hairs (*n* = 2, 4.9%), flame hairs (*n* = 1, 2.4%), bent tapering hairs (*n* = 1, 2.4%), reddish-brown background (*n* = 1, 2.4%), and brown rings around the perifollicular areas (*n* = 1, 2.4%).

## Discussion

The current report represents one of the few on SA available in the literature. This study has included the largest number of patients to date to demonstrate the epidemiology, clinical and trichoscopic features, laboratory results, treatment, and outcomes of SA in the Thai population. We have also performed a systematic review of the literature to define the trichoscopic signs. Our findings revealed that the trichoscopic findings of SA show no specific characteristics and overlap with those of other non-scarring alopecia forms. This diagnostic challenge can be partially overcome by examining the patient for the additional trichoscopic characteristics of SA suggested in our study.

SA is a mucocutaneous manifestation of secondary syphilis in addition to the classic presentations, such as maculopapular eruption, papulosquamous rash predominantly affecting the palms and soles, and condylomata lata (1). However, it can also be the only manifestation of the disease, as demonstrated in the present study. It is important to maintain a high level of diagnostic suspicion to avoid a delayed or an incorrect diagnosis. The prevalence of SA in our study was ~11.9%, which is comparable to that reported previously (1, 2). Men comprised the majority of the patients; this could be because compared to women, men have shorter hair, which allows them to detect hidden scalp lesions more easily. Screening for SA should be performed carefully in women with long hair that may conceal the scalp lesions. Furthermore, human immunodeficiency virus infection was demonstrated in ~50% of the patients with SA; thus, further investigations for sexually transmitted diseases among these populations are recommended (25).

The prevalence of symptomatic and essential SA has not been well-studied. However, it is known that it is uncommon for SA to present with alopecia only. Symptomatic SA was observed more frequently than essential SA in our data and systematic reviews. SA can manifest in three distinct patterns. Moth-eaten SA in the parieto-occipital scalp area was the predominant pattern in our cohort; this is consistent with the findings noted in current literature (3, 14, 26). This pattern is considered the most common type, and is a pathognomonic sign of SA. However, other non-scarring alopecia clinically mimicking moth-eaten alopecia, such as alopecia areata, trichotillomania, and tinea capitis, should be included in the differential diagnoses (2, 9).

Diffuse SA also mimics telogen effluvium, presenting with diffuse hair loss and no obvious patches (10). This is considered a reactive phenomenon, instead of the direct effect of syphilis on the hair follicles (26–28). Nevertheless, some patients with syphilis develop telogen effluvium, which is not the same as diffuse SA (29, 30). Black dots and an erythematous background observed on trichoscopy may help differentiate between the two conditions. In mixed-type SA, both moth-eaten and diffuse patterns can be observed concomitantly. Scarring alopecia is rarely reported in secondary syphilis; however, it has been reported in patients with tertiary syphilis (27). Trichoscopic and histopathological examinations can help in the definitive diagnosis of these cases.

Trichoscopy is increasingly being used for the diagnosis of hair and scalp disorders. Although the specific trichoscopic characteristics of SA have not yet been described, trichoscopy may help in the differential diagnosis of SA. Piraccini et al. also introduced the utility of trichoscopy as a screening tool for SA in patients with secondary syphilis; they noted an increase of 40% in the rate of SA diagnosis, owing to a complete trichoscopic examination of all hair-bearing areas (14). Analysis of our cohort and our systematic review revealed that the majority of the trichoscopic findings of SA were non-specific; several of these findings overlapped with those of other alopecia. These included the zigzag and pigtail hairs in alopecia areata, broken hairs and black dots in tinea capitis and trichotillomania, and empty hair follicles and short regrowing hairs in telogen effluvium (31–33). These findings are consistent with the current literature (34), and SA could also be considered a “great imitator” on trichoscopy. However, the absence of exclamation mark hairs, a trichoscopic hallmark of alopecia areata, may be an important clue to the diagnosis since this feature has never been demonstrated on trichoscopy of SA (20, 34). Further comparative studies with other types of alopecia are required to determine the most useful features.

The pathophysiology of SA remains unclear. SA is hypothesized to be caused by *Treponema pallidum* antigens that induce an immune response (14). Molecular studies have identified the organism in the affected follicles, supporting the theory of specific immunological reactions to treponemal antigens (26–28). Histopathological evidence revealed perivascular and perifollicular lymphocytic dermal infiltrates, and immunohistochemistry confirmed spirochetes to be present in the peribulbar region and penetrate the follicle matrix of SA (26–28, 35). These pathologic findings could explain the trichoscopic features of the hair shaft abnormalities resembling alopecia areata (i.e., zigzag hairs) and trichotillomania (i.e., broken hairs and black dots). Moreover, immune-mediated, small-vessel vasculitis in SA could negatively affect the hair growth cycle and induce reactive phenomena, resulting in trichoscopic changes similar to those in telogen effluvium (i.e., decreased hair per follicular unit, empty follicles, and short regrowing hairs).

Our study indicated that antibiotic treatment for SA is effective in inducing hair regrowth, demonstrating that all patients receiving treatment achieved significant hair regrowth. Therefore, early recognition of mucocutaneous manifestations of syphilis and trichoscopic findings suggestive of SA could facilitate early diagnosis and management, which are crucial for improving treatment outcomes.

The present study incorporates patients with SA from our institute and published articles with relatively adequate case numbers. However, this study has some limitations. Due to its retrospective design, some data may not be available. This study was conducted with a small number of subjects at a single tertiary center; thus, the generalizability of its findings is limited. Some included cases were not biopsy-confirmed; however, this issue could be resolved by the fact that SA in our study was diagnosed based on criteria that were stricter as compared to the criteria in current literature. Data from our systematic review were entirely retrieved from case reports and case series, which limits the methodological quality; the results may be influenced by a subjective interpretation and selective reporting of the trichoscopic features. Further prospective multicenter studies conducted with a larger sample size, including patients with biopsy-proven SA, are recommended to minimize the impact of these limitations and verify our results.

## Conclusion

We have reported the epidemiological characteristics, clinical and trichoscopic features, laboratory results, treatment, and outcomes of Thai patients with SA using the largest case series analyzed to date. SA is an uncommon presentation of secondary syphilis, and physicians should maintain a high level of suspicion, because patients may manifest only alopecia without any mucocutaneous manifestations. Our study also indicates that trichoscopy may facilitate the diagnosis of SA. Because trichoscopy is an increasingly valuable tool for the diagnosis of hair disorders, a systematic review of its reported features may help differentiate SA from other alopecia. Early diagnosis and management of these patients are essential to improve the treatment outcomes.

## Data Availability Statement

The raw data supporting the conclusions of this article will be made available by the authors, without undue reservation.

## Ethics Statement

The studies involving human participants were reviewed and approved by the Mahidol University Institutional Review Board for Ethics in Human Research. Written informed consent for participation was not required for this study in accordance with the national legislation and the institutional requirements.

## Author Contributions

PS: conceptualization and writing-review and editing. PS, CP, and SS: methodology. PS and YR: validation. CP and SS: formal analysis and writing-original draft preparation. PS, CP, SS, and YR: investigation. YR: data curation. All authors have read and agreed to the published version of the manuscript.

## Conflict of Interest

The authors declare that the research was conducted in the absence of any commercial or financial relationships that could be construed as a potential conflict of interest.

## Publisher's Note

All claims expressed in this article are solely those of the authors and do not necessarily represent those of their affiliated organizations, or those of the publisher, the editors and the reviewers. Any product that may be evaluated in this article, or claim that may be made by its manufacturer, is not guaranteed or endorsed by the publisher.
